# Thermal Differences in the Plantar Surface Skin of the Foot after Using Three Different Lining Materials for Plantar Orthotics

**DOI:** 10.3390/life13071493

**Published:** 2023-06-30

**Authors:** Esther Querol-Martínez, Artur Crespo-Martínez, Beatriz Gómez-Martín, Elena Escamilla-Martínez, Alfonso Martínez-Nova, Raquel Sánchez-Rodríguez

**Affiliations:** 1 Clinic Sciences Department, Universitat de Barcelona, 08028 Barcelona, Spain; equerol@ub.edu (E.Q.-M.); arturcrespo@ub.edu (A.C.-M.); 2 Nursing Department, Universidad de Extremadura, 06006 Badajoz, Spain; bgm@unex.es (B.G.-M.); escaelen@unex.es (E.E.-M.); rsanrod@unex.es (R.S.-R.); 3 Centro Universitario de Plasencia, Avda, Virgen del Puerto 2, 10600 Plasencia, Spain

**Keywords:** thermography, orthopedic treatments, materials, orthoses, insoles, foot skin

## Abstract

The lining materials of plantar orthoses are chosen for their hardness, breathability, and moisture absorption, but without there being any clear scientific criterion. Thermographic analysis would provide information about the thermal response of the sole of the foot, and would thereby allow the choice to be adapted in accordance with this criterion. The objective of this study was to evaluate plantar temperatures after the use of three materials with different characteristics. Plantar temperatures were analyzed by using a FLIR E60BX thermographic camera on 36 participants (15 men and 21 women, 24.6 ± 8.2 years old, 67.1 ± 13.6 kg, and 1.7 ± 0.09 m). Measurements were made before and after (3 h) the use of three lining materials for plantar orthoses (Material 1: PE copolymer; Material 2: EVA; Material 3: PE–EVA copolymer) on different days. For Material 1 (PE), the temperature under the heel was significantly higher after exercise, increasing from 30.8 ± 2.9 °C to 31.9 ± 2.8 °C (*p* = 0.008), and negative correlations were found between room temperature and the pre/post temperature difference for the big toe (r = −0.342, *p* = 0.041) and the 1st metatarsal head (r = −0.334, *p* = 0.046). No significant pre/post temperature differences were found with the other materials. The three materials thermoregulated the plantar surface efficiently by maintaining the skin temperature at levels similar to those evaluated before exercise. If PE is used as a lining material, it should be avoided for the heel area in patients with hyperhidrosis or those with a tendency to suffer from skin pathologies due to excess moisture.

## 1. Introduction

Orthopedic insoles are commonly made from a combination of polymeric (usually cellular or foam) materials. On the one hand, there are materials used to manufacture the base of the plantar support, or “shell”, on which the required biomechanical control will be placed. These are mainly polyester resins [1], thermoplastics [2], or foams [3,4]. Another important part in the comfort of the plantar support is the covering or lining material which comes into direct contact with the foot, and thus must have characteristics that prevent the generation of excessive heat, friction, or moisture inside the footwear. A variety of cellular polymers used to manufacture these linings, such as polyethylene (PE), ethyl vinyl acetate (EVA), and others, all of which are of medium or low hardness [4,5,6]. The lining materials on the market are also very diverse with respect to hardness, density, structure (perforated or not), thickness, and thermal, physical, and mechanical properties. The choice is made at the discretion of the prescribing professional in accordance with their clinical experience, the information they receive from the manufacturers (composition, density, thickness, and coefficient of friction), and the cushioning, comfort, or perspiration properties they expect. The reason for this subjectivity is the lack of criteria for the use of these materials due to the limited technical information available concerning them and the lack of scientific evidence that supports their use [4,5,6].

One of the most important characteristics of plantar orthotics related to the thermal response and comfort of polyethylene foams and EVA is their coefficient of friction, which is the ratio between the sliding force and the retention force exerted by two surfaces when in contact. This coefficient is in fact an evaluation of the difficulty with which the surface of one material slides on another material. Thus, greater friction between two such surfaces as the sole of the foot and the lining material could cause a rise in temperature, less breathability, and therefore more sweating. This overheating could lead to patient discomfort, rapid degradation of the materials, and the appearance of skin lesions, such as dermatomycosis or friction vesicles, caused by the high temperature and increased sweating.

A thermographic analysis of the lining materials of foot orthotics could provide quantitative information about the thermal response of the skin of the sole of the foot. Thus, a material could be chosen that is better adapted to the characteristics of the patient and would be more comfortable. Thermal comfort in an orthosis is an important factor that may determine whether it is accepted by the user, so it is understood that materials which generate excessive heating will be less well accepted than those which do not cause such heating. Some clear examples are insoles for sport use and orthoses for patients with diabetes, for whom the temperature of the foot is of vital importance as it can prevent skin lesions resulting from the overheating of the lining materials used [7,8,9,10,11,12]. However, as indicated above, there is little information on this topic. Our working hypothesis is that materials with low friction coefficients will not increase the temperature significantly at the foot sole skin–orthotic lining material interface. The objective of this study was therefore to analyze the different thermographic patterns at the soles of the feet of an adult population after 3 h using three different materials (PE, EVA, and PE–EVA) commonly employed as linings in the manufacture of plantar orthoses.

## 2. Materials and Methods

The sample consisted of 36 participants, 15 men and 21 women, with a mean age of 24.6 ± 8.2 years, a mean weight of 67.1 ± 13.6 kg, and a mean height of 1.7 ± 0.09 m (Table 1). All of them gave their informed consent in compliance with the guidelines and principles of the Declaration of Helsinki, and with approval from the Bioethics and Biosafety Committee of the University of Extremadura (Id:186/2020). The anthropometric characteristics are also listed in Table 1 by sex, with men presenting greater weight, foot size, and height (*p* < 0.001, *p* = 0.002 and *p* < 0.001 respectively).

This study was conducted between January and March 2022. The participants were asked not to engage in intense physical exercise during the 24 h period prior to the measurement, not to consume stimulants such as tobacco, alcohol, tea, or coffee in the preceding 12 h, to avoid the intake of medications or any therapeutic or UV treatments that could affect body temperature, and to avoid eating copious amounts of food and applying cosmetic products to the skin before the test [13,14,15]. The measurements were made on 14 different days, with each participant being measured on 3 different days, 1 for each of the 3 materials tested. The temperature and humidity of the study room were controlled at all times (temperature: 18–20 °C; RH: 40–45%).

The three materials analyzed were of different compositions. For this, an insole of each material was cut for each foot, matching the participant’s foot size, and marked with the participant’s identifier. The technical characteristics of the materials used were as follows:

Material 1: Sidas-Podiatech, Podialene 125. Three millimeters thick and unperforated. PE copolymer expansive foam. Hardness ≈ 25 Shore A. Density ≈ 0.11 g/cm^3^. Medium coefficient of friction. Color: red.

Material 2: nora^®^ Lunatur 27 Walnut. Three millimeters thick and unperforated. EVA. Hardness ≈ 27 Shore A. Density ≈ 0.24 g/cm^3^. Low coefficient of friction. Color: light brown.

Material 3: Sidas-Podiatech. Podiamic 160. Three millimeters thick and unperforated. PE–EVA copolymer expansive foam. Hardness ≈ 35 Shore A. Density ≈ 0.145 g/cm^3^. Low coefficient of friction. Color: skin.

The materials were randomly assigned to each participant in such a way that the participant who was assigned Material 2 on the first day was then assigned Material 3 on the second day and Material 1 on the third day, and so on. In this way, Materials 1, 2, and 3 were analyzed on the same day with different participants, without them knowing the order in which the materials were to be evaluated. One of the researchers was responsible for inserting the insole and removing it from the shoe when necessary. During each session, the measurements of twelve participants were taken. The same protocol was established for all sessions. The following precautions were taken to help the patients prepare for thermal imaging: (1) the feet were thoroughly cleaned; (2) adequate rest was taken before the exam; (3) exposure to direct sunlight was avoided; (4) the application of creams or lotions to the areas to be evaluated was avoided; (5) hot or cold drinks were not consumed before the exam; (6) a stable room temperature was maintained; (7) intense physical activity was avoided before the test; (8) smoking and alcohol consumption were avoided before the test; (9) heating devices were not used on the feet; (10) the application of chemical products to the feet was avoided; and (11) the use of dressings or bandages on the feet was avoided. The participants sat on a stretcher, took off their sanitary shoes, and removed their socks without touching any surface. Their feet were then allowed to acclimatize to the ambient temperature. A black thermal screen was then placed around the ankle zone to prevent the feet from being affected by heat refracted from the rest of the body. The feet were placed in a stable position and any jewelry or metallic adornments were removed. The images were taken in a room with no light reflections and uniform lighting, and the camera was positioned properly with correct focal length and optimized image settings. The thermal images of the plantar area were taken using a FLIR E60BX thermal imaging camera with the following technical characteristics: a resolution of 76.800 pixels, 0.045 °C at 30 °C thermal sensitivity, a temperature range of −20 °C to 120 °C with ±2% or 2 °C accuracy, and a spectral range from 7.5 µm to 13 µm. The camera was placed on a tripod one meter away from the two feet. Once the three thermographic images were taken (emissivity of 0.98, ironbow color palette), the corresponding material was put into the participant’s usual sanitary footwear (each participant had the same brand and model: Medical Shoes Zale^®^, Alicante, Spain). The shoes had a wide toe box, Velcro adjustment at the top, and a heel height of 2.5 cm. The same process was carried out for each of the three materials, and stockings or socks were not worn so as to reduce the post-exercise thermal readjustment time. Next, the participants carried out their usual clinical care activity (3 h duration) which consisted of periods of walking, moments in a sitting position, and others in a static standing position. This activity is representative of a working day in a health clinic in rooms with the same flooring with no unevenness. After these 3 h, the participants returned to the room for the second measurement, which followed the protocol detailed above, and three plantar images were again taken.

**Figure 1:**
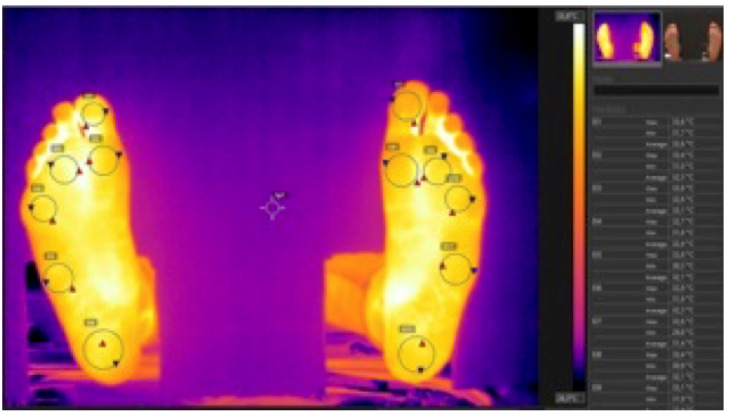
Thermal photograph of a participant’s feet with the analysis of the ROIs.

All the thermographic images were processed using the Flir Tools 6.4 software, and the following six different measurement zones (regions of interest, ROIs) were established on the sole of each foot: the Hallux, the 1st metatarsal head, the 3rd metatarsal head, the 5th metatarsal head, the external arch midpoint (styloid process), and the center of the heel (Figure 1).

### Statistical Analysis

In order to maintain data independence [16], all the variables analyzed corresponded to the participant’s right foot, this being chosen at random. The mean temperature of each zone was calculated from the three plantar images taken for each lining material before and after the physical exercise. After verifying that the sample data fitted normality (Kolgomorov–Smirnov test, *p* > 0.05 in all cases), (1) a Student’s *t*-test for paired samples was performed to verify the pre/post temperature differences, and (2) Pearson correlation coefficients were calculated to identify whether the temperature in the room influenced the participants’ plantar temperatures. Since the temperature data also met the assumption of sphericity (*p* > 0.05 in each of the three-layer comparisons), a repeated measures ANOVA was carried out (3 × 3 with Bonferroni confidence model adjustment) for the temperature differences (pre/post) for each of the three materials. Statistical analyses were performed using SPSS version 22.0 (UEX campus license), and the significance level was set at 5% (*p* < 0.05).

## 3. Results

For the PE copolymer expansive foam (Material 1), the temperature in the plantar zone of the 3rd metatarsal head was 31.2 ± 3.0 °C before exercise, while after exercise it was 31.9 ± 2.9 °C, though the difference was not statistically significant (*p* = 0.159). Heel temperature was significantly higher after exercise, increasing from 30.8 ± 2.9 °C to 31.9 ± 2.8 °C (*p* = 0.008) (Table 2). For the EVA foam (Material 2), the temperature in the plantar zone of the 3rd metatarsal head was 31.9 °C before exercise, while after exercise it was 32.1 °C, though the difference was not statistically significant (*p* = 0.655). At the styloid process, a temperature of 31.7 °C was observed before exercise, and a temperature of 31.9 °C was observed after exercise (*p* = 0.748, Table 2). The pre/post thermal differences were not significant in any of the zones analyzed (*p* > 0.05 in all cases). For the PE–EVA copolymer expansive foam (Material 3), the temperature in the plantar zone of the 1st metatarsal head was 30.9 °C before exercise, while after exercise it was 31.3 °C, though the difference was not statistically significant (*p* = 0.299). In the zone of the 5th metatarsal head, a temperature of 30.6 °C was observed before exercise, and a temperature of 31.1 °C was observed after exercise (*p* = 0.247, Table 2). The pre/post thermal differences were not significant in any of the zones analyzed (*p* > 0.05 in all cases, Table 2).

When testing Material 1 (PE copolymer), negative correlations were found between room temperature and the temperature differences (pre/post) under the big toe (*p* = 0.041) and the 1st metatarsal head (*p* = 0.046). However, no correlations were found between room temperature and the foot temperatures for the other two materials (EVA and PE–EVA). When comparing the temperature variations (pre/post) between the three materials, no significant differences (*p* > 0.05 in all cases) were observed in any of the zones analyzed (Table 3). However, there seemed to be a tendency for Material 2 (EVA) to present a lower temperature under the big toe after exercise, since the variation was −0.15°C (*p* = 0.051, Table 3).

## 4. Discussion

This study aimed to analyze foot sole temperatures after 3 h of exercise using three different materials (PE, EVA, PE–EVA) commonly used as linings in the manufacture of plantar orthoses. For Material 1 (PE), it was observed that there is a tendency for the temperature to increase by +0.6 °C to +0.8 °C in the midfoot and forefoot zones, with the only statistically significant increase being in the heel zone (+1.1 °C). For Materials 2 and 3, the variations found were from −0.2 °C to +0.3 °C (EVA) and from +0.1 °C to 0.7 °C (PE–EVA), although these were not statistically significant. These results seem to indicate that all three materials can efficiently regulate foot temperature, keeping it at levels similar to those recorded before exercise.

The significant temperature increase in the heel zone for Material 1 (PE) could be because, during human gait, the contact of the heel with the ground is the biomechanical moment of greatest impact, and this zone together with those of the metatarsal heads are subject to the most pressure and friction during walking. In addition to this, such characteristics as the coefficient of friction and even the color may have influenced this increase in temperature. Material 1 (PE) was the only material with a medium coefficient of friction, while Materials 2 (EVA) and 3 (PE–EVA) had low coefficients of friction.

Thus, it could be concluded that materials with low coefficients of friction might be cooler and thermoregulate more efficiently, and thus be better as plantar orthosis linings (as is indicated by the variations in temperature). Currently, companies specializing in the sale and distribution of materials for the manufacture of orthopedic insoles are researching new and more sustainable raw materials of plant origin to avoid the increase in temperature generated around the feet of users. Since these studies are not independent and have not been conducted over extended periods, there is still a great deal to be learned in this field. 

Another potential factor to take into account is the color of the material used, since Material 1 (PE) was red and was the darkest of the three materials, the other two being light brown (Material 2, EVA) and the color of skin (Material 3, PE–EVA). Chromatism could be a factor affecting the generation of a higher temperature in the foot, since red or pink colors absorb more light and heat than lighter colors [17,18,19,20]. In addition, it seems that, in the zone under the big toe, the temperature variation for Material 2 (EVA) had a tendency (*p* = 0.051) to be negative, i.e., for the temperature to be cooler after exercise. Other external factors such as ambient temperature and humidity can influence thermal differences in the plantar area. The foot is exposed to environmental conditions, so when the ambient temperature is low, heat transfer from the foot to the colder environment is possible, and when the ambient temperature is high, the plantar surface temperature is more likely to increase due to exposure to high temperatures and a lack of perspiration. When the environment is humid, sweat is more likely to accumulate on the foot and on the insole cladding material, which is in direct contact with the foot and acts as an additional thermal insulator, and this can cause the temperature of the plantar surface to increase, affecting the feeling of comfort.

The negative correlation between the temperature in the room where the measurements were taken (within 18–20°) and the temperatures under the big toe and the 1st metatarsal head indicated that the higher the room temperature was, the lower these two measured temperatures would be. Since there is no plausible biomechanical explanation for this, it reinforces the idea that the coefficient of friction or the color of the material could have influenced these results (though they may nonetheless have been coincidental).

Before carrying out this study, we believed that gait patterns could be affected by thermal differences in the plantar surface of the foot if these were high enough to produce dermal lesions that altered the biomechanics of the foot. However, the comparison between the temperature increases of the three materials has shown us that, zone by zone, there are no differences between the temperature increases of the three materials used. A zone-by-zone comparison of the temperature increases of the three materials (Table 3) revealed no differences. Results such as those reported by Gil-Calvo et al. [21] reinforce the theory that the type of material used has no influence on plantar temperatures. Their work analyzed subjects after an intense run in three situations: with their own shoe, and with plantar supports made using two different lining materials (Drytech polyurethane and PE–EVA). They found that the use of insoles did not affect plantar temperature, but that plantar temperature increased for the participants who ran in their own shoes without insoles. Jiménez-Pérez et al. [22] reached similar conclusions in their study, in which two different insoles were used in a run, one with a polyurethane lining with carbon and the other with a polyester lining. They observed no plantar temperature changes and concluded that the insole did not influence the temperature. These results, together with those of the present work, show that linings can have a thermoregulatory effect on the sole of the foot since they maintain temperature and avoid excessive heat being generated during physical activity.

In general, the variations in plantar temperature that the present study’s participants experienced after performing physical activity were very small compared with those reported in other studies—increases of 3 °C have been observed following short (10 min) walks [23]; increases of 7 °C have been observed in medium-intensity runs [21], and increases of 10–14 °C have even been observed in high-intensity runs [22]. This is because the activities carried out by our subjects were discontinuous and of low intensity since they were intended to be representative of their usual clinical care activity, i.e., periods of walking, sitting, resting, etc. It would be of great interest to carry out a new comparative study analyzing the thermographic behavior of these three materials with the same subjects but with different levels of physical activity and different durations of activity so as to observe their thermal properties and their involvement in different walking patterns.

A possible limitation of this study is that the temperature was evaluated with only the lining material, and not with the orthosis shell material whose type (resin, thermoplastics, etc.) might well have had an influence. In addition, the participants were tested without socks to prevent their composition from influencing their foot temperatures. Though the number of steps would certainly affect heat distribution, this was not recorded; the use of a pedometer could therefore have provided new insights. Another limitation was the duration of the study, which was three months. It was therefore not possible to study the long-term effects of the coating materials used on thermal regulation, or their relationship with the general health of the foot. On the other hand, according to the studies of Lo et al. [9] and García De La Peña et al. [11], we know that if we take into account the relevance of temperature in diabetic patients, for whom coating materials are closely associated with moisture absorption performance and thermal comfort, important factors in the prevention of ulcerations may be revealed [24,25].

## 5. Conclusions

EVA, PE, and PE–EVA foams are effective at thermoregulating the sole of the foot during the performance everyday activities. The use of dark-colored polyethylene (PE) foam or materials with medium-to-high coefficients of friction should be avoided in the heel zone in subjects with potential sweating problems or with a tendency to develop skin lesions.

## Figures and Tables

**Table 1:** Anthropometric characteristics of the participants and differences by sex.

		Mean	SD	*p*
Age		24.6	8.2	
	Men	26.9	12.1	0.160
	Women	23.0	3.1	
Shoe Size		40.4	3.0	
	Men	43.4	1.5	<0.001
	Women	38.2	1.4	
Weight		67.2	13.6	
	Men	75.0	13.4	0.002
	Women	61.6	10.9	
Height		1.7	0.1	
	Men	1.8	0.1	<0.001
	Women	1.6	0.1	

**Table 2:** Paired sample statistics for the feet. Material 1: PE copolymer; Material 2: EVA foam; Material 3: PE–EVA copolymer expansive foam.

	Mean	SD	DIF.	*p*
PE copolymer expansive foam (Material 1)				
Hallux pre Hallux post	29.8	3.6	0.7	0.244
	30.5	3.6		
1st MTH pre 1st MTH post	30.9	2.9	0.5	0.243
	31.5	2.9		
3rd MTH pre 3rd MTH post	31.2	3.0	0.7	0.159
	31.9	2.9		
5th MTH pre 5th MTH post	30.5	2.9	0.7	0.103
	31.3	2.9		
Styloid process pre Styloid process post	31.1	2.3	0.6	0.125
	31.7	2.5		
Heel pre Heel post	30.8	2.9	1.1	0.008
	31.9	2.8		
EVA foam (Material 2)				
Hallux pre	30.9	3.5	−0.2	0.761
Hallux post	30.7	3.6		
1st MTH pre	31.7	2.8	−0.1	0.947
1st MTH post	31.6	2.8		
3rd MTH pre	31.9	2.8	0.2	0.655
3rd MTH post	32.1	2.9		
5th MTH pre	31.3	2.8	0.1	0.852
5th MTH post	31.4	2.8		
Styloid process pre	31.7	2.5	0.2	0.748
Styloid process post	31.9	2.4		
Heel pre	31.5	2.5	0.3	0.426
Heel post	31.8	2.5		
PE–EVA copolymer expansive foam (Material 3)				
Hallux pre	30.0	3.0	0.5	0.299
Hallux post	30.5	3.7		
1st MTH pre	30.9	2.6	0.4	0.385
1st MTH post	31.3	3.2		
3rd MTH pre	31.3	2.4	0.4	0.342
3rd MTH post	31.7	3.1		
5th MTH pre	30.6	2.4	0.5	0.247
5th MTH post	31.1	3.1		
Styloid process pre	31.4	2.2	0.1	0.849
Styloid process post	31.4	2.8		
Heel pre	31.1	2.4	0.7	0.101
Heel post	31.8	2.9		

**Table 3:** Comparison of the temperature variations of the three materials.

	M1	M2	M3	W Mauchly (Sig)	Pillai’s Trace	*p*
	Mean °C					
Hallux	0.68	−0.15	0.49	0.974 (*p* = 0.644)	0.051	0.414
1st MTH	0.54	−0.02	0.35	0.963 (*p* = 0.522)	0.035	0.542
3rd MTH	0.67	0.18	0.41	0.966 (*p* = 0.557)	0.024	0.664
5th MTH	0.72	0.07	0.50	0.976 (*p* = 0.662)	0.042	0.479
Styloid process	0.61	0.11	0.07	0.968 (*p* = 0.573)	0.043	0.573
Heel	1.1	0.27	0.67	0.974 (*p* = 0.644)	0.080	0.242

## Data Availability

Data are available in http://www.unex.es/investigacion/grupos/biopiex, accessed on 17 May 2023.
